# Viral Hepatitis in Mongolia: Past, Present, and Future

**DOI:** 10.5005/jp-journals-10018-1168

**Published:** 2016-07-09

**Authors:** Bira Tsatsralt-Od

**Affiliations:** National Institute of Medicine, Ministry of Health and Ministry of Science Education Mongolia, Ulaanbaatar, Mongolia

**Keywords:** Hepatitis A virus, Hepatitis B virus, Hepatitis C virus, Hepatitis D virus, Hepatitis E virus.

## Abstract

Viral hepatitis is one of the major health concerns worldwide, particularly in Asian countries. Mongolia, which is located in northern Asia, between Russia and China, is confronting various infectious diseases, such as viral hepatitis and tuberculosis. As for healthy individuals in Mongolia, the reported prevalence of hepatitis B surface antigen (HBsAg) was 9 or 10% and the reported prevalence of anti-hepatitis C virus ranged from 11 to 25%. We reported a markedly high prevalence of hepatitis D virus RNA (83%) among apparently healthy individuals with HBsAg in Ulaanbaatar. Also due to lack of proper mechanisms to handle sewerage, disinfection, and lack of clean water supply across the country, hepatitis A is endemic in Mongolia. Moreover, Mongolia ranked in the high-prevalence zone for hepatitis B, D, and C.

**How to cite this article:**

Tsatsralt-Od B. Viral Hepatitis in Mongolia: Past, Present, and Future. Euroasian J Hepato-Gastroenterol 2016;6(1):56-58.

## HEPATITIS A AND E INFECTION IN MONGOLIA

Infection with hepatitis A virus (HAV) is one of the common causes of liver disease of great public health importance in the world. However, the level of endemicity, the median age at the time of infection, and the frequency of clinically apparent hepatitis caused by HAV vary by population.^1^ The seroprevalence rate of HAV is highly correlated with the socioeconomic status and access to clean water and sanitation. Mongolia, one of the developing countries in Asia, has been considered to be highly endemic for HAV infection. However, there have been little or no data on the serological and molecular epidemiology of HAV infection among children in Mongolia. The overall prevalence of anti-HAV among apparently healthy persons aged 0 to 20 years was found to be 68.9%, and the age dependency of HAV seroprevalence was noted. All but 1 of the 15 neonates 7 to 28 days of age had anti-HAV. The mother of the infant who was negative for anti-HAV was 19 years old, and the absence of anti-HAV in this infant may have been due to the lack of anti-HAV in the maternal antibodies (i.e., lack of anti-HAV in cord blood and colostrum). The prevalence of anti-HAV decreased to 72.7% in the age group 1 to 2 months, 50% in the age group 3 to 4 months, and 25.0% in the age group 5 to 6 months, and reached 0% in infants aged 7 to 11 months, suggesting the gradual disappearance of passively transferred maternal antibody against HAV within 6 months of life.^2^

Then, the prevalence of anti-HAV increased from 1 to 20 years of age, being 19.5% in the age group 1 to 3 years, 50% in the age group 4 to 6 years, and > 80% in the age group 7 to 9 years. Nearly all subjects (97.2%) in the age group 16 to 20 years had anti-HAV.^3^ This is in agreement with our previous observation that all 249 apparently healthy adults aged 23 to 86 years were immune against HAV.^4^

Subgenotype IA HAV was recovered from all 3 children with current infection with HAV, confirming results from a previous study that subgenotype IA was prevalent among patients with hepatitis A in Mongolia.^2^ The Mongolian HAV isolates were more closely related over the entire genome to those circulating in China and Japan. The seroprevalence of two hepatitis viruses, HAV and HEV, transmitted enterically were compared. Hepatitis A virus and HEV had different epidemiological patterns in the Mongolian children, and only 5 children (0.7%) were found to be positive for anti-HEV IgG.^2^ The very low prevalence of HEV infection among children found in this study is in contrast to a study in the adult population in which as high as 11% were positive for anti-HEV IgG.^4^ In this year we have revealed the occurrence of auhochthonous patients first time with acute hepatitis E in Mongolia, caused by a monophyletic genotype 4 strain.^5^

## HEPATITIS B, D, AND C INFECTION IN MONGOLIA

Hepatitis B virus (HBV) is a blood-borne and sexually transmitted virus that is acquired by percutaneous and mucosal exposure to blood or other body fluids of an infected person. Clinical manifestations of acute hepatitis B can be severe, and serious complications (i.e., cirrhosis and liver cancer) are more likely to develop in chronically infected persons.

Since 1952, viral hepatitis has been reported officially, and since 1981, acute hepatitis B has been reported separately. Hepatitis B vaccine was introduced into the EPI in 1991. However, since 1991, the incidence of acute hepatitis B has declined steadily. Seroprevalence of hepatitis B surface antigen (HBsAg) in the unvaccinated population has been 11 to 29%, slightly higher compared with other countries.^3^ In 1991, the Hepatitis B vaccine was introduced into routine immunization schedule. To date, the coverage of this vaccine has reached more than 96% and the prevalence of HBV among vaccinated children from 1993 to 2000 decreased to 3.5%, according to a meta-analysis.^6^ MOH and NCCD implemented the “Improvement of Hepatitis B Vaccine Immunization Program in Mongolia” with the support of UNICEF and WHO in 2005–2006. The outcome of the project was increased birth dose coverage of hepatitis B vaccine within 24 hours after born. Also we should strengthened the vaccine transfortation system and vaccine storage especially in the rural areas. The nationwide survey “Impact Assessment of National Vaccination Program against Hepatitis B in Mongolia-2” in 2009–2010 showed that the prevalence of HBV carriage significantly decreased by 0.53% among vaccinated children aged 4 to 6 years. An analysis of long-term dynamics of hepatitis B indicates the incidences of HBV were 8.9 and 12.2 per 10,000 populations in age groups 15 to 19 and 20 to 24 years in 2006–2009 and increased by 2.5 and 2.1 times respectively, compared to 1998–2000. Hence we need to assess the immunity level against HBV today and decide to provide supplementary Hepatitis B vaccination to the risk group. Prevalence study among the 403 blood donors were detected; 33 (8.2%), 21 (5.2%), and 27(6.7%) tested positive for HBsAg and/or HBV DNA, hepatitis C virus (HCV) RNA, and hepatitis D virus (HDV RNA) respectively. Our previous study revealed high endemicity for HBV, HCV, and HDV infections among apparently healthy individuals.^7^ However, there are little or no data on the prevalence and genotype distribution of HBV, HCV, and HDV among patients with chronic liver disease in Mongolia. Therefore, serum samples obtained in 2004 from 207 patients including those with chronic hepatitis were tested for serological and molecular markers of HBV, HCV, and HDV infections. Of the 207 patients, 144 (69.6%), 106 (51.2%), and 117 (56.5%) tested positive for HBsAg and/or HBV DNA, HCV RNA, and HDV RNA respectively. Collectively, 172 patients (83.1%) were viremic for one or more of these viruses, including dual viremia of HBV/HDV (26.6%) or HBV/HCV (7.7%) and triple HBV/HCV/HDV viremia (30.0%).^8^ Of note, triple ongoing infection was significantly more frequent among patients with hepatocellular carcinoma than among those with chronic hepatitis (63.2% *vs* 14.4%, p < 0.0001). One hundred sixty patients (77.3%) had a history of blood transfusion and/or surgery. The distribution of HBV genotypes among the 116 HBV-viremic patients was: A (0.9%), B (0.9%), C (6.0%), D (88.8%), and C plus D (3.4%). All 117 HDV isolates were classified into genotype I. The 106 HCV RNA-positive samples were categorized as genotype 1b (92.5%), 2a (0.9%), or 1b plus 2a (6.6%); mixed infection of two distinct HCV genotypes was found exclusively in patients with hepatocellular carcinoma.^8^

## CONCLUSION

The burden of five types of viral hepatitis and its complications is a huge challenge for the government and health care providers in rural areas in Mongolia. The future strategy has four parts and the first one reflects hepatitis A. Measures to prevent viral hepatitis A have been taken since 1972 as a social determinant according to the resolutions and orders of the government and the MOH. The strategy recommends to administer viral hepatitis A vaccine according to the schedule as reflected and implemented in the national immunization program in stages. Vaccine against HAV was introduced in 2012 by the National Center for Communicable Diseases. The second part describes viral hepatitis B issue. The viral hepatitis B morbidity has declined as a result of measures such as single-use syringe for healthcare and service and scheduled immunization of newborn babies with hepatitis B vaccine since 1991. But given the evidence of high risk of hepatitis B among professionals such as hospital staff and high morbidity of viral hepatitis B among citizens born before 1991 under the survey, a proposal has been developed to immunize these groups of population with hepatitis B vaccine. The third part elaborates on viral hepatitis C. At present there is no hepatitis C vaccine internationally. Mongolia is considered to have high spread of viral hepatitis C, so proposals to introduce modern diagnostic methods and technology for the disease to health care and services and provide the state support accordingly have been made in the viral hepatitis C strategy. Also it has been deliberated as necessary to improve compliance with infection control and disinfection procedure by hospitals and nonmedical organizations (cosmetic such as tattooing and ear piercing) to prevent viral hepatitis B, C, and D that transmit parenterally, introduce modern disinfection technology, and improve capacity. The next part is about the diagnosis and treatment of viral hepatitis. The innovative side of the part lies in changing traditional methods of treatment and diagnosis of viral hepatitis particularly viral hepatitis C and proposing to use modern antiviral drugs, such as harmony and sofosbuvir. Also diagnostic and treatment guidelines for viral hepatitis found in pregnant women and guidance on home treatment of the viral hepatitis A have been reviewed and redeveloped differently at each level of health care and service.
